# Alteration of Autonomic Nervous System Is Associated With Severity and Outcomes in Patients With COVID-19

**DOI:** 10.3389/fphys.2021.630038

**Published:** 2021-05-19

**Authors:** Yuchen Pan, Zhiyao Yu, Yuan Yuan, Jiapeng Han, Zhuo Wang, Hui Chen, Songyun Wang, Zhen Wang, Huihui Hu, Liping Zhou, Yanqiu Lai, Zhen Zhou, Yuhong Wang, Guannan Meng, Lilei Yu, Hong Jiang

**Affiliations:** ^1^Department of Cardiology, Renmin Hospital of Wuhan University, Wuhan, China; ^2^Cardiac Autonomic Nervous System Research Center of Wuhan University, Wuhan, China; ^3^Cardiovascular Research Institute, Wuhan University, Wuhan, China; ^4^Hubei Key Laboratory of Cardiology, Wuhan, China

**Keywords:** COVID-19, heart rate variability, autonomic nervous system, clinical outcomes, d-dimer, lymphocyte, N-terminal Pro-B-type natriuretic peptide

## Abstract

**Background:**

Previous studies suggest that coronavirus disease 2019 (COVID-19) is a systemic infection involving multiple systems, and may cause autonomic dysfunction.

**Objective:**

To assess autonomic function and relate the findings to the severity and outcomes in COVID-19 patients.

**Methods:**

We included consecutive patients with COVID-19 admitted to the 21st COVID-19 Department of the east campus of Renmin Hospital of Wuhan University from February 6 to March 7, 2020. Clinical data were collected. Heart rate variability (HRV), N-terminal pro-B-type natriuretic peptide (NT-proBNP), D-dimer, and lymphocytes and subsets counts were analysed at two time points: nucleic-acid test positive and negative. Psychological symptoms were assessed after discharge.

**Results:**

All patients were divided into a mild group (13) and a severe group (21). The latter was further divided into two categories according to the trend of HRV. Severe patients had a significantly lower standard deviation of the RR intervals (SDNN) (*P* < 0.001), standard deviation of the averages of NN intervals (SDANN) (*P* < 0.001), and a higher ratio of low- to high-frequency power (LF/HF) (*P* = 0.016). Linear correlations were shown among SDNN, SDANN, LF/HF, and laboratory indices (*P* < 0.05). Immune function, D-dimer, and NT-proBNP showed a consistent trend with HRV in severe patients (*P* < 0.05), and severe patients without improved HRV parameters needed a longer time to clear the virus and recover (*P* < 0.05).

**Conclusion:**

HRV was associated with the severity of COVID-19. The changing trend of HRV was related to the prognosis, indicating that HRV measurements can be used as a non-invasive predictor for clinical outcome.

## Introduction

Coronavirus disease 2019 (COVID-19) is a newly recognised infectious disease, and has rapidly spread worldwide. As of November 1, 2020, more than 40,000,000 cases have been confirmed throughout the world. The death toll has risen dramatically, with more than 1,000,000 deaths having been recorded. Early identification and intervention were crucial for reducing mortality.

The severity of COVID-19 is evaluated according to vital symptoms, physical signs, and physiological parameters. Although COVID-19 is primarily manifested as respiratory disease, many studies have shown that it is a systemic infection involving multiple systems. Previous studies have attempted to predict the severity and prognosis based on immune function, myocardial injury, and coagulation (Chen et al., 2020; Guo et al., 2020; Zhou et al., 2020). The autonomic nervous system plays an important role in the regulation of the whole-body homeostasis, including immune system, the cardiovascular system, the hematological system, and so on (van Westerloo et al., 2006; Kenney and Ganta, 2014; Goldberger et al., 2019). It has been proposed that monitoring vagal tone in patients with COVID-19 as a predictive marker of COVID-19 illness course (Bonaz et al., 2020). However, the relationship between autonomic nervous function and disease severity and clinical outcomes of COVID-19 pneumonia has not yet been well delineated. Heart rate variability (HRV) analysis is a reliable tool indicating autonomic modulation and has been widely used as a non-invasive method. In this study, we aimed to assess the ability of HRV measures to indicate the severity and prognosis of COVID-19.

## Materials and Methods

### Study Design and Participants

In this study, we enrolled 34 consecutive patients admitted to the 21st COVID-19 Department of the east campus of Renmin Hospital of Wuhan University, Wuhan, China, from February 6 to March 7, 2020. This designated hospital is responsible for the treatment of severe COVID-19 patients. The diagnosis of all patients was based on the Diagnosis and Treatment of New Coronavirus Pneumonia (trial version seven)^1^; according to the guidelines, 13 of them were classified as mild patients (laboratory confirmed, without pneumonia), and rest were severe patients (dyspnoea, respiratory frequency ≥ 30/min, blood oxygen saturation ≤ 93%, PaO_2_/FiO_2_ ratio < 300, and/or lung infiltrates > 50% of the lung field within 24–48 h). This study was reviewed and approved by the Medical Ethical Committee of Renmin Hospital of Wuhan University (approval number WDRY2020-K079).

### Data Collection

Detailed clinical data for all 34 patients were collected from electronic medical records by physicians who worked at Renmin Hospital of Wuhan University during the epidemic period, including demographic characteristics (sex and age), clinical symptoms and signs, comorbidities, laboratory examination results (routine blood parameters, coagulation function, myocardial injury markers, lymphocyte subpopulation, etc.), treatment and clinical outcome. Recovery was defined by discharge from the hospital. Viral RNA negative conversion was defined by negative results of at least two consecutive tests for SARS-CoV-2 nucleic acids during hospitalization. Severe patients also had a follow-up telephone call monthly after discharge by trained investigators. And psychological symptoms were assessed by the 2-item Generalized Anxiety Disorder Scale (GAD-2) and the Patient Health Questionnaire-2 (PHQ-2). The end of follow-up was July 26, 2020, 3 months from the last discharge. All the data were entered into the electronic database after deleting patients’ private information.

### Electrocardiogram Recording and HRV Analysis

A Holter monitor (Synwing Tech, Chengdu, China) was used to record dynamic electrocardiogram (ECG) data over 24 consecutive hours for all patients. The data were collected into the electronic database, including the time, duration, subsection, count, and the kind of arrhythmia. Then, the HRV was analysed according to the 24 h dynamic ECG recordings. All the data calculated automatically by the computer were manually adjusted and verified by experienced physicians. The intervals with significant changes were excluded to prevent the effect of data mistakes. Time domains were calculated from 24 h dynamic ECG recordings including standard deviation of the RR intervals (SDNN, normal values: 141 ± 39 ms), standard deviation of the averages of NN intervals (SDANN, normal values: 127 ± 35 ms), square root of the mean of the sum of the squares of differences between adjacent NN interval (RMSSD, normal values: 27 ± 12 ms), and percent differences between adjacent NN intervals that are greater than 50 ms (pNN50, normal values: 16.7 ± 12.3%). Frequency-domain analysis included low-frequency power 0.04–0.15 Hz (LF, normal values: 300–1,750 ms^2^), high-frequency power 0.15–0.4 Hz (HF, normal values: 50–120 ms^2^), and the ratio of low- to high-frequency power (LF/HF, normal values: 1–3).

### Statistical Analysis

All categorical variables were compared by using the Pearson Chi-Square test or Fisher’s exact test, and all continuous variables were compared by using an unpaired t-test, a paired *t*-test, the Mann–Whitney test, or the Wilcoxon signed rank test, as appropriate. Categorical data are expressed as proportions. Continuous data are expressed by using the median (25th percentile-75th percentile). Receiver operating characteristic (ROC) curve analysis was used to evaluate the predictive ability of HRV parameters. Kaplan–Meier curves were used to analyse patient prognosis. Pearson rank correlation analysis and Spearman rank correlation analysis were used to determine the correlation. All statistical analyses were processed by SPSS 26.0 (IBM, Chicago, IL, United States), Prism 8 (GraphPad), and MedCalc (version 19.2.1). P values < 0.05 were considered significant.

## Results

### Clinical Characteristics of COVID-19 Patients

The research population included consecutive hospitalised patients who were laboratory confirmed to be positive for COVID-19: 13 patients (38%) were classified as mild patients, and 21 patients (62%) were classified as severe. The mean age was 56.2 ± 16.0 years old, and 23 (68%) were female. Compared with mild patients, severe patients were older (*P* < 0.05). Evaluation was performed on HRV at two time points (nucleic acid test positive and negative). The initial HRV corresponded to the positive status of the nucleic acid test and the follow-up HRV corresponded to the negative status of the nucleic acid test. The time elapsed since admission to initial HRV and follow-up HRV, and the mean interval between the initial and follow-up HRV were significantly shorter in the mild group compared with the severe group (*P* < 0.001, *P* < 0.001, *P* = 0.001, respectively). Similarly, the time elapsed since admission to nucleic acid test positive (corresponding to initial HRV) and nucleic acid test negative (corresponding to follow-up HRV) were significantly shorter in the mild group compared with the severe group (*P* < 0.001, *P* < 0.001, respectively). Among these patients, fever [26 patients (76%)] was the most common symptom. Cough, shortness of breath, fatigue, diarrhoea, and chest pain were present in 15 patients (44%), 5 patients (15%), 7 patients (21%), 3 patients (9%), and 5 patients (15%), respectively. Of the 34 patients, 10 (30%) had comorbidities. Of these 34 patients, 9 (26.47%) had comorbidities. Hypertension [5 patients (14.71%)] and coronary heart disease [2 patients (5.88%)] were the most common comorbidities. Treatment mainly included antiviral therapy (100%), antibiotic therapy (67.65%), traditional Chinese medicine treatment (91.18%), and gut microbiota regulation (82.35%) (Table 1).

**TABLE 1:** Clinical characteristics of all patients.

Variable	Total	Mild group	Severe group	*P*-value
		
	(*n* = 34)	(*n* = 13)	(*n* = 21)	
Sex (male), *n* (%)	11 (32%)	3 (23%)	8 (38%)	0.465
Age, median, year	56.2 ± 16.0	47.5 ± 14.2	61.5 ± 15.0	0.011
Hospital length of stay	24 (15.8–39.3)	15 (13–16.5)	36 (26.5–47)	<0.001
Time from onset to discharge	46 (32.8–52.3)	38 (22.5–45.5)	50 (39–58)	0.002
Days to initial HRV^†^	8.5 (6.8–19)	7 (5–7)	17 (8.5–24.5)	<0.001
Days to nucleic acid test positive (corresponding to initial HRV) ^†^	9.5 (6.8–20)	7 (5.5–8)	18 (9.5–25.5)	<0.001
Days to follow-up HRV^†^	18 (11.8–26.8)	12 (11–14)	26 (18–37)	<0.001
Days to nucleic acid test negative (corresponding to follow-up HRV) ^†^	17 (11–26.8)	11 (10–12.5)	25 (17.5–34)	<0.001
Days between initial and follow-up HRV	7 (6–12.5)	6 (4–7)	10 (7–14)	0.001
**Signs and symptoms, *n* (%)**				
Fever	26 (76 %)	9 (69%)	17 (81%)	0.679
Cough	15 (44%)	7 (54%)	8 (38%)	0.484
Shortness of breath	5 (15%)	1 (8%)	4 (19%)	0.627
Fatigue	7 (21%)	2 (15%)	5 (24%)	0.682
Diarrhoea	3 (9%)	1 (8%)	2 (10%)	1.000
Chest pain	5 (15%)	2 (15%)	3 (14%)	1.000
**Comorbidities**				
Hypertension	5 (14.71%)	3 (23.08%)	2 (9.52%)	0.348
Diabetes	1 (2.94%)	1 (7.69%)	0	0.382
Coronary heart disease	2 (5.88%)	1 (7.69%)	1 (4.76%)	1.000
Cerebrovascular disease	1 (2.94%)	0	1 (4.76%)	1.000
**Treatment**				
Empirical antiviral therapy	34 (100%)	13 (100%)	21 (100%)	1.000
Empirical antimicrobial therapy	23 (67.65%)	8 (61.54%)	15 (71.43%)	0.709
Traditional Chinese medicine treatment	31 (91.18%)	13 (100%)	18 (85.71%)	1.000
Gut microbiota regulator	28 (82.35%)	11 (84.62%)	17 (80.95%)	1.000

*^†^Days elapsed since admission.*

### HRV Results in Patients With Different Severities of COVID-19

**FIGURE 1:**
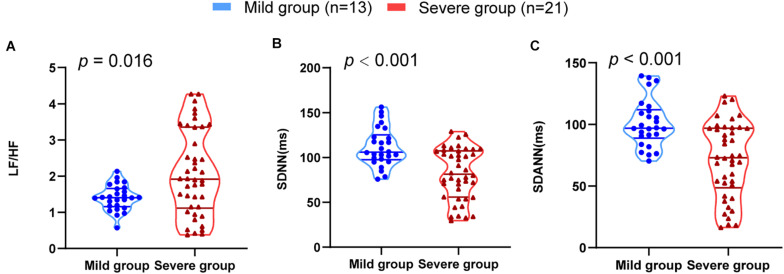
HRV measurements in mild and severe patients with COVID-19. **(A)** LF/HF **(B)** SDNN **(C)** SDANN are shown.

The results of HRV indices were consistent with the severity of illness. The severe group had a significantly lower SDNN (*P* < 0.001) and SDANN (*P* < 0.001) and a higher LF/HF than the mild group (*P* = 0.016) (Figure 1). All other variables showed no significant difference between the groups.

**FIGURE 2:**
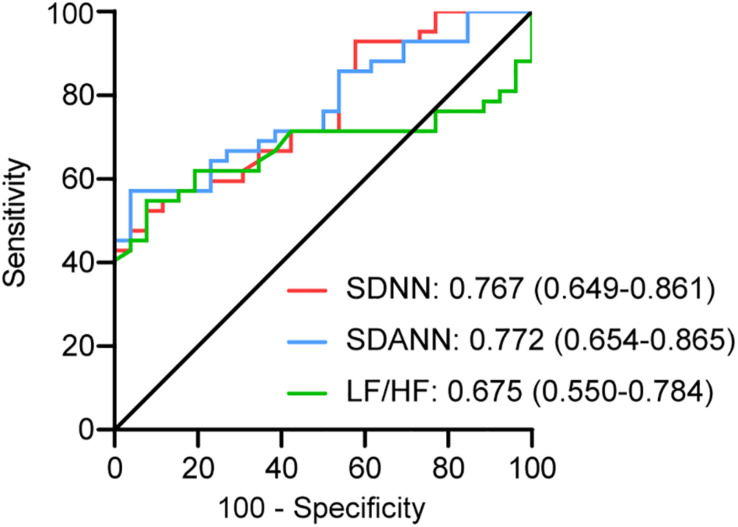
ROC analysis for significant HRV variables. The sensitivity and specificity of SDNN, SDANN, and LF/HF for the severity of COVID-19.

To assess the diagnostic value of the SDNN, SDANN, and LF/HF, ROC curve analysis was used. The ROC curve was above the diagonal, indicating good sensitivity and specificity. The areas under the curve (AUCs) were 0.767 (95% CI, 0.649–0.861), 0.772 (95% CI, 0.654–0.865), and 0.675 (95% CI, 0.550–0.784), respectively, in severe patients with COVID-19 (Figure 2).

### The Correlations Between HRV and Immune, Coagulation, and Myocardial Injury

**FIGURE 3:**
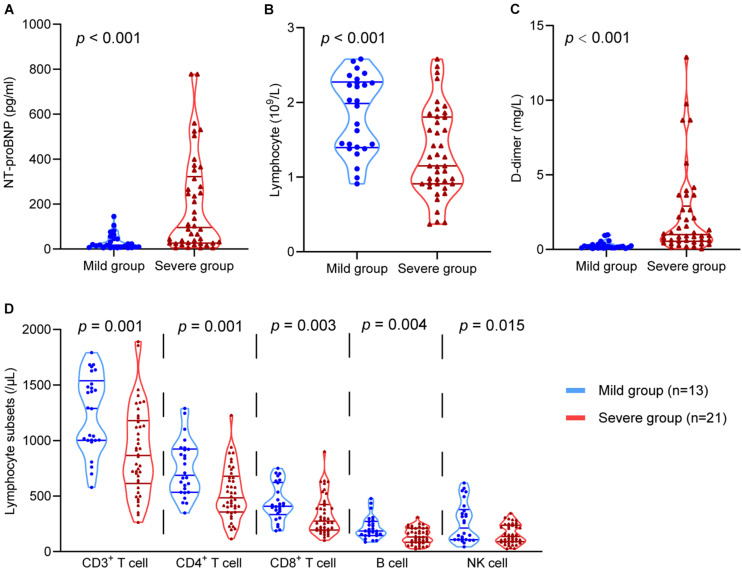
The comparison of the D-dimer, NT-proBNP, lymphocytes, and lymphocyte subsets in the mild group and the severe group. The comparison of **(A)** NT-proBNP, **(B)** lymphocytes, **(C)** D-dimer, **(D)** CD3^+^ T cells, CD4^+^ T cells, CD8^+^ T cells, B cells, and NK cells in different groups.

Lymphocytes and lymphocyte subsets, NT-proBNP, and D-dimer were used to reflect immune function, cardiac function, and coagulation function, respectively. These indicators at the same time point as the HRV test were analysed. The levels of D-dimer (*P* < 0.001) and NT-proBNP (*P* < 0.001) in severe patients were much higher than those in mild patients. The levels of lymphocytes (*P* < 0.001), CD3^+^ T cells (*P* = 0.001), CD4^+^ T cells (*P* = 0.001), CD8^+^ T cells (*P* = 0.003), B cells (*P* = 0.004), and NK cells (*P* = 0.015) in severe patients were significantly lower than those in mild patients (Figure 3). These data suggest that immune, coagulation, and cardiac function are associated with disease severity.

**FIGURE 4:**
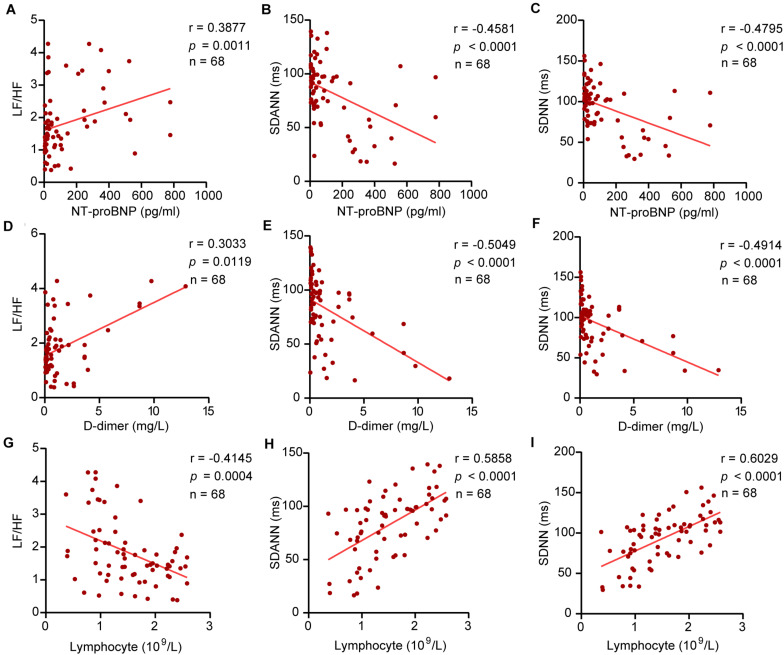
The correlation between HRV and NT-proBNP, D-dimer, and lymphocytes. **(A)** LF/HF and NT-proBNP, **(B)** SDANN and NT-proBNP, **(C)** SDNN and NT-proBNP, **(D)** LF/HF and D-dimer, **(E)** SDANN and D-dimer, **(F)** SDNN and D-dimer, **(G)** LF/HF and lymphocytes, **(H)** SDANN and lymphocytes, **(I)** SDNN and lymphocytes.

Our study revealed the relationship between HRV and D-dimer, NT-proBNP, and lymphocytes. The results showed that NT-proBNP was negatively correlated with SDANN (*r* = –0.4581, *P* < 0.0001) and SDNN (*r* = –0.4795, *P* < 0.0001) and positively correlated with LF/HF (*r* = 0.3877, *P* = 0⋅0011) in COVID-19 patients. Similarly, D-dimer showed correlations with SDANN (*r* = –0.5049, *P* < 0.0001), SDNN (*r* = –0.4914, *P* < 0.0001), and LF/HF (*r* = 0.3033, *P* = 0.0119). The lymphocyte count also had a linear correlation with SDANN (*r* = 0.5858, *P* < 0.0001), SDNN (*r* = 0.6029, *P* < 0.0001), and LF/HF (*r* = –0.4145, *P* = 0.0004) in COVID-19 patients (Figure 4). Similar results were obtained in CD3^+^ T cells, CD4^+^ T cells, and CD8^+^ T cells (Supplementary Figure 1). However, there was no significant correlation between SDNN and B cells, LF/HF and NK cells (Supplementary Figure 2). Our study also correlated HRV to the level of TNF-α. The results showed that TNF-α was negatively correlated with SDANN (*r* = –0.6680, *P* < 0.0001) and SDNN (*r* = –0.2767, *P* = 0.224) and positively correlated with LF/HF (*r* = 0.4109, *P* = 0.0005) in COVID-19 patients (Supplementary Figure 3).

### The Relationship Between the Changing Trend of HRV and Outcomes of COVID-19

**FIGURE 5:**
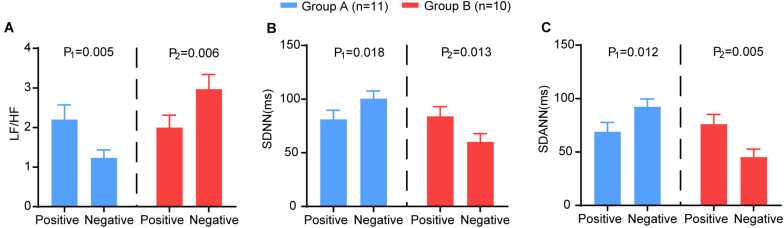
The comparison of the HRV variables between the two time points (nucleic acid test positive and negative) in group A (blue) and group B (red). The **(A)** LF/HF, **(B)** SDNN, **(C)** SDANN in different groups. P-Values were provided for comparison as follows: P1: Nucleic acid test positive vs nucleic acid test negative for group A. P2: Nucleic acid test positive vs nucleic acid test negative for group B.

We analysed the changing trend of HRV between the two time points (nucleic acid test positive and negative) in severe patients. We further subdivided the patients into two groups according to the trend of LF/HF: Group A (LF/HF decreased, *n* = 11) and Group B (LF/HF increased, *n* = 10). Group A showed significantly increased SDNN (*P* = 0.018) and SDANN (*P* = 0.012) and decreased LF/HF (*P* = 0.005). In group B, the opposite trend was observed: the SDNN (*P* = 0.013) and SDANN (*P* = 0.005) decreased significantly, and the LF/HF (*P* = 0.006) increased significantly (Figure 5). Other HRV indices exhibited no differences between the two time points. There was no significant difference between the two groups at baseline (Supplementary Table 1).

**FIGURE 6:**
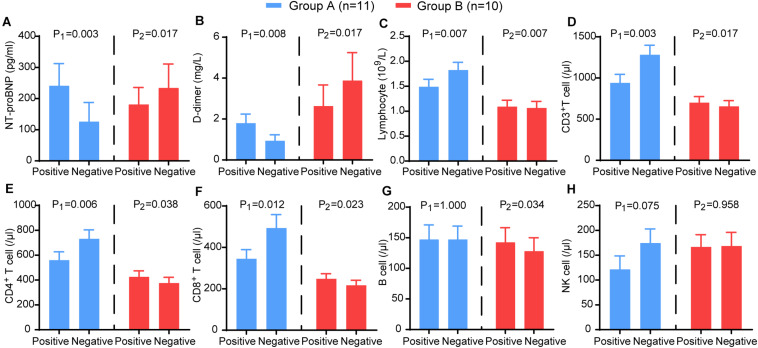
The comparison of the laboratory examination variables between the two time points (nucleic acid test positive and negative) in group A (blue) and group B (red). The **(A)** NT-proBNP, **(B)** D-dimer, **(C)** CD3+ T cells, **(D)** CD4+ T cells, **(E)** CD8+ T cells, **(F)** B cells, **(G)** NK cells, **(H)** lymphocytes in different groups. P-values were provided for comparison as follows: P1: Nucleic acid test positive vs nucleic acid test negative for group A. P2: Nucleic acid test positive vs nucleic acid test negative for group B.

Furthermore, we analysed the trends of NT-proBNP, D-dimer, lymphocytes and lymphocyte subsets. In group A, NT-proBNP (*P* = 0.003) and D-dimer (*P* = 0.008) showed a significant decrease, and lymphocytes (*P* = 0.007), CD3^+^ T cells (*P* = 0.002), CD4^+^ T cells (*P* = 0.006), and CD8^+^ T cells (*P* = 0.012) showed a significant increase. In contrast, NT-proBNP (*P* = 0.017) and D-dimer (*P* = 0.007) increased in group B, and lymphocytes (*P* = 0.007), CD3^+^ T cells (*P* = 0.016), CD4^+^ T cells (*P* = 0.038), CD8^+^ T cells (*P* = 0.023), and B cells (*P* = 0.034) decreased significantly (Figure 6). These results suggest that HRV may be useful as a non-invasive method reflecting the trends of immune function, coagulation, and myocardial injury in patients with COVID-19.

**FIGURE 7:**
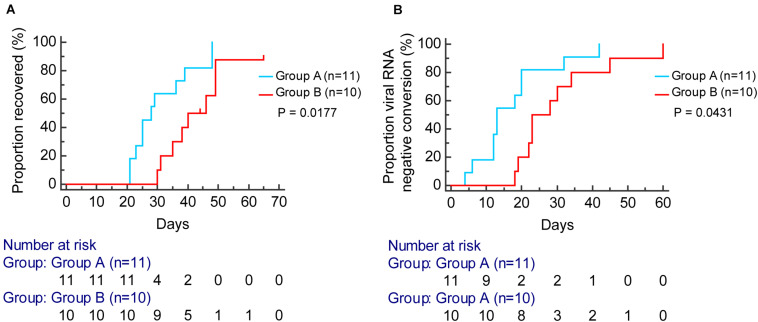
Kaplan–Meier curve analysis for the proportion of viral RNA negative conversion **(A)** and recovered **(B)** stratified by group A (blue) and group B (red).

According to the Kaplan–Meier analysis, patients in group A had a shorter time to viral RNA negative conversion [median, 13 days, compared with 25 days; hazard ratio (HR), 2.36; 95% confidence interval (CI), 0.94–5.90; *P* = 0.0431] and recovery of disease (median, 28 days, compared with 42 days; HR: 3.42; 95% CI, 1.24–9.46; *P* = 0.0177) than patients in group B (Figure 7).

The severe patients were followed up for three months after discharge from the hospital. There was no positive RNA test after discharge in severe patients. Clinical features such as fever, cough, didn’t recur. However, 47, 21, and 26% of patients, respectively, still presented fatigue, shortness of breath, or chest stuffiness after exercise. About 32% of patients reported anxiety symptoms, and 11% reported depression symptoms (Supplementary Table 2).

## Discussion

**FIGURE 8:**
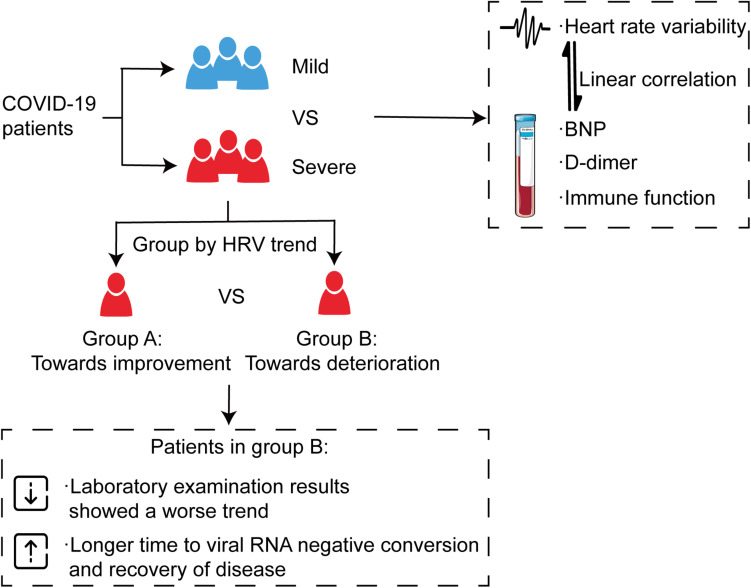
Alteration of autonomic nervous system is associated with severity and outcomes in patients with COVID-19. All patients were divided into a mild group and a severe group, and the severe group was then further divided into two categories according to the trend of HRV indices, which showed a consistent trend with immune function, D-dimer, and NT-proBNP. The results showed that severe patients without improved HRV parameters needed a longer time to clear the virus and recover.

In this study, we tried to predict severity and outcome with autonomic changes in patients with COVID-19. Besides, this study demonstrated novel evidence that HRV was associated with the severity of the disease. Indeed, severe patients tended to show more severe impaired HRV, which exhibited a linear correlation with NT-proBNP, D-dimer, and immune function. Based on these findings, in the next step, we further investigated the effect of HRV changing trend on outcomes of severe patients. The results showed that severe patients without improved HRV parameters needed a longer time to clear the virus and recover, indicating that HRV measurements can be used as a non-invasive predictor for clinical outcome (Figure 8).

The autonomic nervous system plays a critical and complex role in maintaining the body’s balance (Ulrich-Lai and Herman, 2009). Time-domain and frequency-domain analysis of the HRV is often used as a non-invasive, simple, and effective tool to evaluate autonomic function. Research has found that viral infection can affect HRV measurements. Chronic fatigue syndrome patients with Epstein-Barr virus (EBV) show higher plasma adrenaline and norepinephrine levels and lower LF/HF in HRV (Kristiansen et al., 2019). The variables of baroreflex sensitivity and HRV in patients with hepatitis C virus were also found to be lower than those in healthy patients (Osztovits et al., 2009). In a cross-sectional study included 325 participants, HIV-infected participants had lower HRV than HIV-uninfected participant (Godijk et al., 2020). Moreover, previous studies have shown that HRV alters in the presence of systemic infection, and HRV monitoring may enhance the diagnosis and prognosis of infection, and HRV may help evaluate risk of deterioration in patients with sepsis and merits validation and further evaluation (Ahmad et al., 2009; Barnaby et al., 2019). However, there is no previous study focusing on HRV measures in predicting severity and outcomes in patients with COVID-19.

Our research showed that severe patients had more severe autonomic dysfunction than mild patients, as indicated by the HRV analysis. Our results showed lower SDNN and SDANN and higher LF/HF in the severe group than in the mild group. The ROC curve illustrated the significant discriminatory power of these indices. This study was limited by a small sample size, which may explain the lack of statistical significance in other HRV indices, such as RMSSD and PNN50. Our results agreed with previous findings that COVID-19 patients with a more serious degree of disease or a poor outcome had more pronounced decreases in immune parameters, higher cardiac injury biomarkers, and more severe coagulation dysfunction than patients with milder disease (Chen et al., 2020; Guo et al., 2020; Zhou et al., 2020). Some studies have shown that COVID-19 contributes to arrhythmias (Guo et al., 2020; Wang et al., 2020), and similar findings have been noted for SARS and MERS (Yu et al., 2006; Saad et al., 2014), which may eventually be due to autonomic nerve function changes.

The autonomic nervous system is closely related to many pathophysiological processes. Neurotransmitters produced by autonomic nerves interact with immune cells, including neutrophils, monocytes, and T cells, to regulate immunoreactions and inflammation (Pavlov et al., 2018). The correlation between autonomic nerve function and NT-proBNP, TNF-α has already been proposed (Kyuma et al., 2004; Pellissier et al., 2014). Furthermore, the present study showed that these interesting correlations exist between HRV and NT-proBNP, D-dimer, and immune function in COVID-19. These laboratory indicators have already been proven to be closely related to disease conditions (Chen et al., 2020; Guo et al., 2020; Zhou et al., 2020). These findings indicated that HRV is correlated with disease severity and may be used in monitoring disease conditions and estimating prognosis. Our study further confirmed the association between the trend of HRV and outcome. We analysed the trend of HRV between nucleic acid test positive and negative in severe patients during hospitalization. Severe patients with an improvement in autonomic parameters showed improvements in immune function, coagulation function, and cardiac injury biomarkers. Consistent with previous studies, the decline in and recovery of immune function in patients with COVID-19 were dominated by T cells (Chen et al., 2020). Furthermore, Kaplan-Meier analysis showed that patients without improved HRV parameters needed a longer time to viral RNA negative conversion and recovery.

Studies have shown that some patients with a detectable positive RNA test after discharge (Hoang et al., 2020; Ye et al., 2020). Therefore, the patients were followed up from admission to three months after discharge. There was no recurrence of positive SARS-CoV-2 in patients recovered from COVID-19. However, our results showed 47, 21, and 26% of patients, respectively, still presented fatigue, shortness of breath, or chest stuffiness after exercise. About 32% of patients reported anxiety symptoms, and 11% reported depression symptoms.

The cause for the more severe autonomic dysfunction in the severe group is not clear and might be multifactorial. Lung injury leading to hypoxemia, which can affect autonomic nerve activity (Gao et al., 2015), might be one of the mechanisms. The virus can also directly cause myocardial cell damage or aggravate pre-existing myocardial conditions, which may induce conduction disturbances (Akhmerov and Marban, 2020). Additionally, COVID-19 causes anxiety and depression in patients, which is related to autonomic dysfunction (Hu et al., 2018).

Previous studies indicated that controlled cytokine release is imperatively linked to a well-balanced autonomic nervous system (Tracey, 2002). It has been reported that the main injury COVID-19 caused is the cytokine storm, which is known as a continuously ineffective inflammatory response (De Virgiliis and Di Giovanni, 2020). In this study, our results suggested that autonomic balance and vagal nerve stimulation should be taken into consideration when evaluating diagnostic and therapeutic approaches of COVID-19. In other disease patterns related to uncontrolled cytokine release, VNS has been used as a part of the therapeutic approach (Merrill et al., 2006; Bonaz et al., 2013, 2016; Koopman et al., 2016). Alternatively, it has also been proposed that Beta-adrenergic blockers decrease the renin level by their inhibitory action on the sympathetic system, which may produce beneficial effects in COVID-19 patients (Natesan Vasanthakumar, 2020).

Evaluating the host response to SARS-Cov-2 infection as a complex system provides novel insights for predicting. Compared with other laboratory indicators, HRV is a widely accepted, noninvasive method for the evaluation of autonomic balance. Moreover, HRV-evaluation is cost-neutral and available for use under study- and clinical conditions. Our findings are preliminary because they are based on a single-center study with a small sample of patients with COVID-19. However, ECG recordings were carefully acquired and analysed to reduce errors introduced by the experimental method. Although confirming the predictive ability of HRV requires larger validation studies, this pilot study presents the first data to suggest that HRV may be a non-invasive marker for COVID-19.

## Conclusion

This study suggests that patients with COVID-19 with autonomic dysfunction are more likely to have an increased severity of illness. The underlying mechanism of these findings for the prognosis of COVID-19 is unclear. However, this knowledge about HRV as a predictor of severity and outcome is important for monitoring disease progression and assessing treatment effects. Further study recruitment may shed more light on the predictive ability of HRV. There is no single tool for diagnosis or prediction in COVID-19. As a non-invasive modality, HRV biomarkers can be combined with other clinical predictors to monitor disease conditions and estimate prognosis, which may avoid alternative medical prescription and intervention. Besides, HRV may also guide the treatment of patients with autonomic dysfunction. Vagal tone restore therapy may help rebalance the autonomic nervous system of patients, which shows a potential application value in COVID-19 treatment.

## Data Availability Statement

The raw data supporting the conclusions of this article will be made available by the authors, without undue reservation.

## Ethics Statement

The studies involving human participants were reviewed and approved by this study was reviewed and approved by the Medical Ethical Committee of Renmin Hospital of Wuhan University (approval number WDRY2020-K079). Written informed consent for participation was not required for this study in accordance with the national legislation and the institutional requirements.

## Author Contributions

HJ and LY conceptualised the study. YP, ZY, YY, and JH designed the study. YP, YY, ZhuW, HC, SW, ZheW, and HH recruited patients and collected and processed samples. ZY, JH, LZ, YL, ZZ, YW, and GM performed the statistical analyses. YP, ZY, YY, and JH co-wrote the manuscript. All authors read and approved the final version of the manuscript.

## Conflict of Interest

The authors declare that the research was conducted in the absence of any commercial or financial relationships that could be construed as a potential conflict of interest.

## Footnotes

1 The Diagnosis and Treatment of New Coronavirus Pneumonia (trial version seven), National Health Commission of China, New coronavirus pneumonia prevention and control program (7th edn). http://www.nhc.gov.cn/yzygj/s7653p/202003/46c9294a7dfe4cef80dc7f5912eb1989/files/ce3e6945832a438eaae415350a8ce964.pdf. (5 May 2020).

## Abbreviations

HRV: heart rate variability
NT-proBNP: N-terminal pro-B-type natriuretic peptide
SDNN: standard deviation of the RR intervals
SDANN: standard deviation of the averages of NN intervals
RMSSD: square root of the mean of the sum of the squares of differences between adjacent NN interval
pNN50: percent differences between adjacent NN intervals that are greater than 50 ms
LF: low-frequency power
HF: high-frequency power
LF/HF: ratio of low- to high-frequency power
GAD-2: 2-item Generalized Anxiety Disorder Scale
PHQ-2: Patient Health Questionnaire-2
ECG: electrocardiogram
ROC: receiver operating characteristic.
