# Supplementary material for: Alteration of Autonomic Nervous System Is Associated With Severity and Outcomes in Patients With COVID-19
Source: Front Physiol. 2021 May 19;12:630038. doi: 10.3389/fphys.2021.630038 (PMC8170133; doi:10.3389/fphys.2021.630038)
Supplement: Supplementary file 1 [file Data_Sheet_1.pdf]

Supplemental figure legends

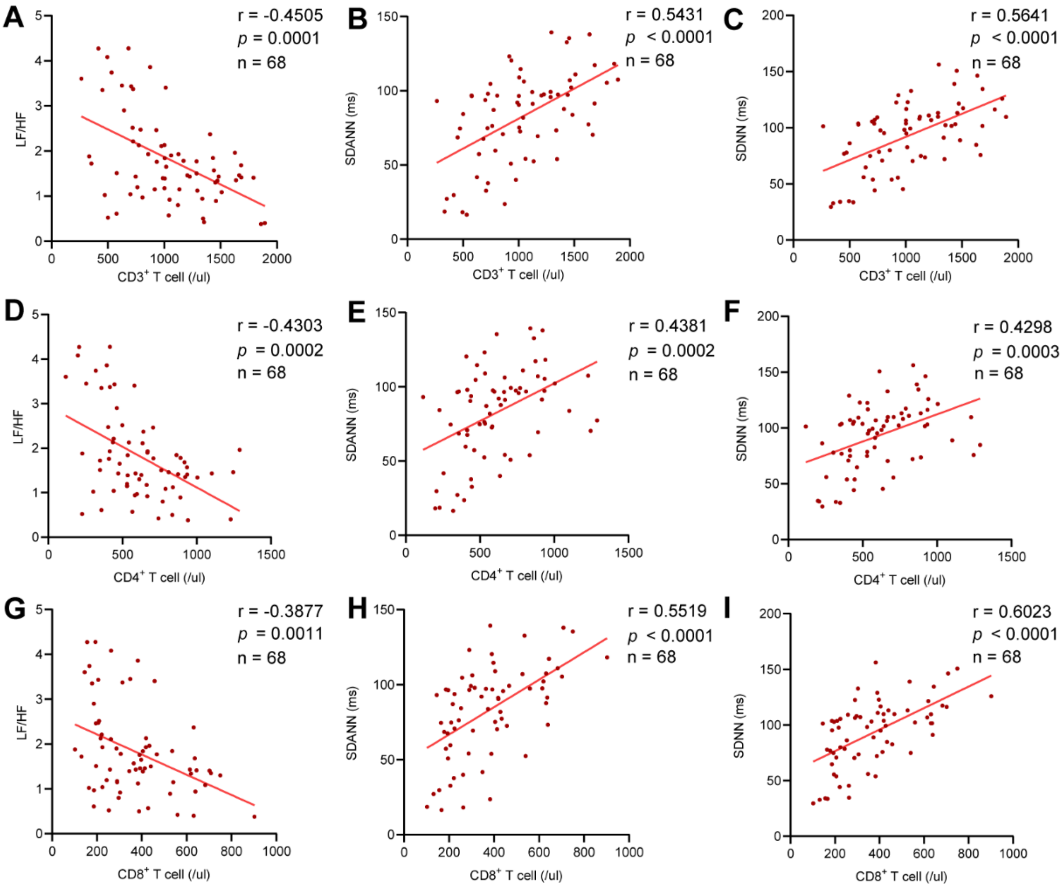

Figure S1. The correlation between HRV and CD3+ T cell, CD4+ T cell, and CD8+ T cell. (A) LF/HF and $CD3^+$ T cell, (B) SDANN and $CD3^+$ T cell, (C) SDNN and $CD3^+$ T cell, (D) LF/HF and $CD4^+$ T cell, (E) SDANN and $CD4^+$ T cell, (F) SDNN and $CD4^+$ T cell, (G) LF/HF and $CD8^+$ T cell, (H) SDANN and $CD8^+$ T cell, (I) SDNN and $CD8^+$ T cell.

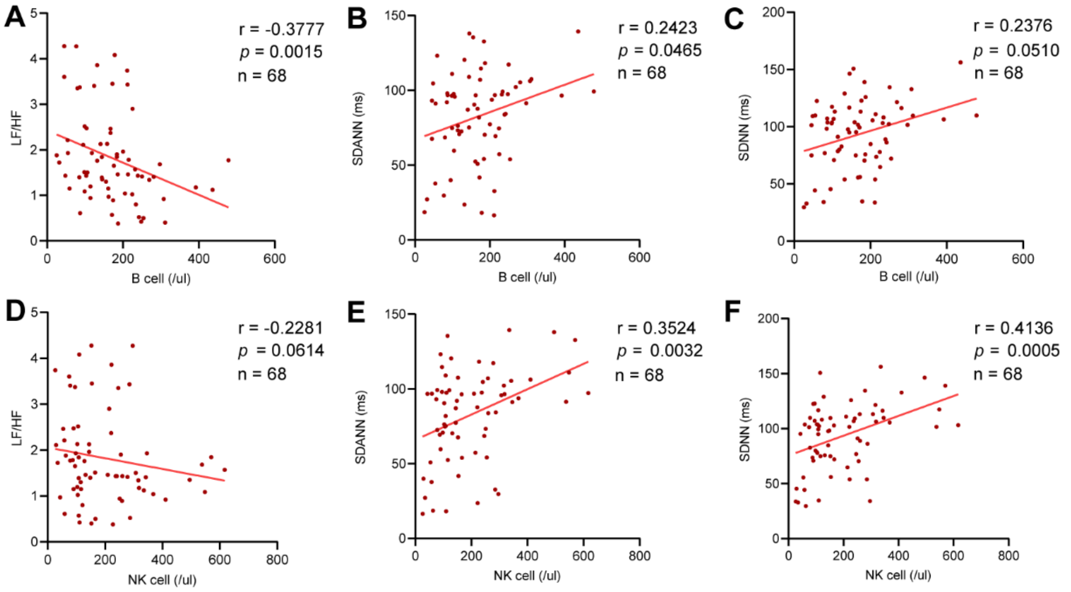

Figure S2. The correlation between HRV and B cell, NK cell. (A) LF/HF and B cell, (B) SDANN and B cell, (C) SDNN and B cell, (D) LF/HF and NK cell, (E) SDANN and NK cell, (F) SDNN and NK cell.

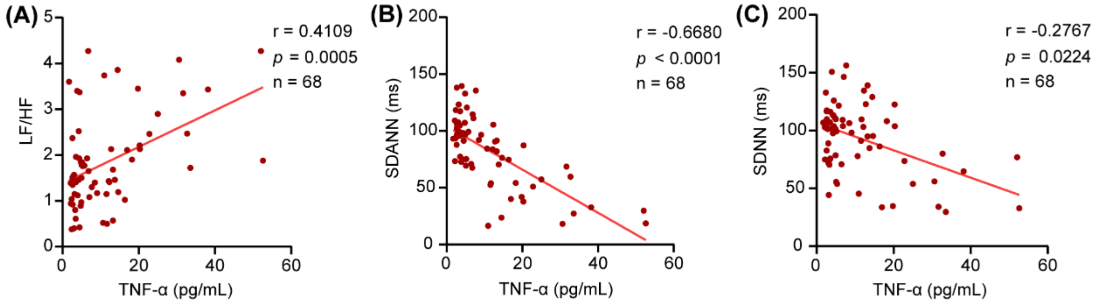

Figure S3. The correlation between HRV and TNF-α. (A) LF/HF and TNF-α, (B) SDANN and TNF-α, (C) SDNN and TNF-α.

Supplemental Table 1

Clinical characteristics and baseline in group A and B

	Group A (n = 11)	Group B (n = 10)	*P* value
Sex (male), *n* (%)	4(36%)	4(40%)	1.000
Age, year	54.0(48.0-62.0)	62.5(59.3-72.0)	0.111
Comorbidities	2(18%)	2(20%)	1.000
Initial HRV			
LF, 300 ~ 1750, ms^2	188.3(74.7-319.1)	207.3(106.4-516.2)	0.360
HF, 50 ~ 120, ms^2	97.7(33.7-276.4)	134.1(63.8-210.0)	0.968
LF/HF, 1 ~ 3	2.2(1.2-3.4)	1.8(1.3-3.0)	0.679
SDNN, 140±39, ms	82.7(55.5-107.1)	83.1(62.5-108.4)	0.827
SDANN, 127±35, ms	72.6(50.9-96.8)	77.6(53.4-100.5)	0.584
SDNN Index, 37±15, ms	37.7(27.5-43.0)	35.2(28.2-42.3)	0.856
RMSSD, 127±35, ms	15.2(10.7-18.7)	19.5(12.0-28.4)	0.481
pNN50, 16.7±12.3, %	0.6(0.1-1.5)	2.9(0.2-6.7)	0.360
Laboratory examinations			
NT-proBNP, 0~900, pg/ml	165.1(66.1-372.8)	165.1(66.1-372.8)	0.517
D-dimer, 0~0.55, mg/L	0.9(0.6-3.6)	1.1(0.6-4.2)	0.888
Lymphocyte, 1.1~3.2, 10^9/L	1.4(0.9-1.7)	1.0(0.9-1.4)	0.310
$CD3^+$ T cell, 723-2737, /μL	1012.0(723.0-1205.0)	694.5(488.5-876.8)	0.082
$CD4^+$ T cell, 404-1612, /μL	580.0(414.0-742.0)	410.5(324.8-488.8)	0.127
$CD8^+$ T cell, 220-1129, /μL	388.0(195.0-457.0)	238.5(184.0-309.8)	0.076
B cell, 80-616, /μL	161.0(55.0-213.0)	136.5(72.0-227.3)	0.891
NK cell, 84-724, /μL	95.0(58.0-166.0)	149.0(115.3-242.3)	0.091

Supplemental Table 2

Follow-up results after discharge in severe patients

	No.(%)			*P* value
	Total (n = 19)	Group A (n = 11)	Group B (n = 8)	
Viral reactivation	0	0	0	-
Signs and symptoms				
Fever	0	0	0	-
Cough	0	0	0	-
Fatigue	9(47%)	3(27%)	6(75%)	0.070
Shortness of breath after exercise	4(21%)	1(9%)	3(38%)	0.262
Chest stuffiness after exercise	5(26%)	1(9%)	4(50%)	0.111
Anxiety	6(32%)	2(18%)	4(50%)	0.319
GAD-2 score †	2.0(2.0-3.0)	2.0(1.0-2.0)	2.5(2.0-3.0)	0.068
Depression	2(11%)	1(9%)	1(13%)	1.000
PHQ-2 score‡	1.0(1.0-2.0)	1.0(0.0-2.0)	1.5(1.0-2.0)	0.128

†GAD-2 consists of the first two items of the GAD-7, scores ≥ 3 reflecting anxiety symptoms

‡PHQ-2 consists of the first 2 items of the PHQ-9, scores ≥ 3 reflecting depression symptoms
